# Comment on "Dectin-1 is Pathogenic in Chronic Kidney Disease by Promoting Macrophage Infiltration and Transition to Myofibroblast"

**DOI:** 10.7150/ijbs.125175

**Published:** 2026-01-29

**Authors:** Dequan Liu, Xiangyu Che, Guangzhen Wu

**Affiliations:** Department of Urology, the First Affiliated Hospital of Dalian Medical University, Dalian, 116011, China.

The study "Dectin-1 is Pathogenic in Chronic Kidney Disease by Promoting Macrophage Infiltration and Transition to Myofibroblast," published in the International Journal of Biological Sciences 1 brings to light the important role of Dectin-1, a pattern recognition receptor, in the progression of chronic kidney disease (CKD), particularly its involvement in macrophage infiltration and transition to myofibroblasts, both key events in the development of renal fibrosis.

Macrophage-myofibroblast transformation (MMT) is a lineage switch in which bone marrow-derived macrophages acquire myofibroblast characteristics and directly contributes to extracellular matrix deposition in chronically injured kidneys 2, 3. MMT cells are often identified by dual macrophage/myofibroblast markers within the fibrotic microenvironment and have been confirmed in human CKD biopsies and multiple mouse models 2, 3. The authors have presented a comprehensive analysis, beginning with a clear correlation between Dectin-1 expression and kidney fibrosis in CKD patients. This work offers new insights into the complex immune and fibrotic processes associated with CKD. The use of a range of experimental models, including UUO, IR, and bone marrow chimeric mice, to validate their findings enhances the strength of their conclusions. Particularly noteworthy is the detailed mechanistic exploration of Dectin-1's role in macrophage infiltration via the Syk/NF-κB/CCL2-CCR2 axis, and its facilitation of macrophage-myofibroblast transition (MMT) through the TGF-β/Smad pathway (Figure 1).

The authors highlight the role of Dectin-1 in macrophage infiltration and activation. However, the study does not fully distinguish how Dectin-1 affects different macrophage subtypes. For instance, what Figure 6 shows is that knocking out Dectin-1 depresses both iNOS and CD206 frequencies and MFIs, a broad activation deficit. That does not preclude a bias toward M1 in WT; it just means the KO dampens activation across the board at this timepoint. M1 and M2 macrophages play contrasting role in inflammation and fibrosis. M1 macrophages are classically activated by signals such as IFN-γ or LPS and are characterized by their pro-inflammatory functions, including the production of cytokines like IL-1β, IL-6, and TNF 4, 5. Conversely, M2 macrophages are alternatively activated by IL-4/IL-13 and contribute to tissue repair, remodeling, and anti-inflammatory responses through mediators such as IL-10 and TGF-β, with macrophages in vivo often displaying phenotypic plasticity along an M1-M2 continuum in response to local microenvironmental cues 6. Exploring whether Dectin-1 influences the balance between these subtypes could provide more nuanced therapeutic insights (Figure 1). Meanwhile, as the authors demonstrate here, the current studies focus on demonstrating that Dectin-1 promotes fibrosis in models of angiotensin II, UUO, and ischemia-reperfusion, primarily through Syk/NF-κB-dependent CCL2/CCR2 chemokine recruitment and TGF-β/Smad-driven MMT 1, 7. While these data establish Dectin-1 as a driver of renal inflammation and profibrotic programs, the interplay with CTGF or Wnt/β-catenin has not been specifically investigated. Notably, Dectin-1-induced β-catenin activation has been demonstrated in non-renal immune settings, supporting its biological plausibility, but has not been validated in the kidney 8, 9. Given the central role of CTGF and the Wnt/β-catenin pathway in renal fibrosis 10, 11, the authors could conduct targeted experiments to determine whether Dectin-1 interacts synergistically with these pathways in the renal environment, potentially leading to a deeper understanding of the molecular network underlying renal fibrosis. The use of pharmacological inhibitors targeting key mediators of these pathways or genetic models may help refine the proposed mechanisms. Combined with the above research content, we can have a more complete understanding of the important clinical application value of Dectin-1 as a predictive biomarker for fibrosis progression or treatment response.

In conclusion, this study provides an important contribution to our understanding of Dectin-1's role in CKD progression and renal fibrosis. I believe that further investigation into the macrophage-specific and broader modulatory roles of Dectin-1, as well as clinical validation of the findings, will be essential in paving the way for Dectin-1-targeted therapies in CKD management.

## Figures and Tables

**Figure 1:**
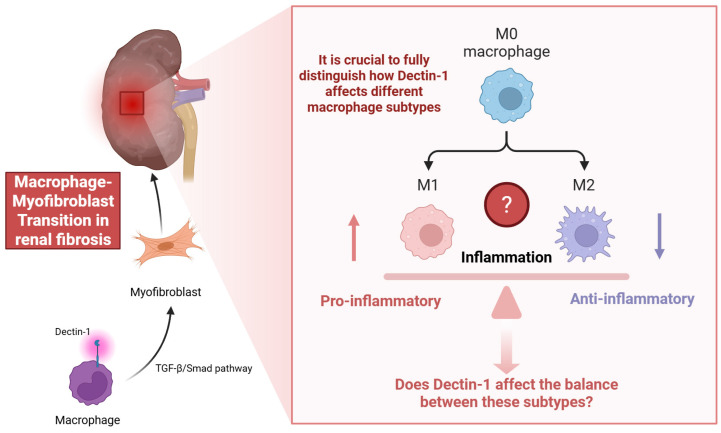
Macrophage-myofibroblast transition in renal fibrosis.

## References

[1] Shen L, Li J, Zhang A, Yan S, Sha W, Wang Y (2025). Dectin-1 is Pathogenic in Chronic Kidney Disease by Promoting Macrophage Infiltration and Transition to Myofibroblast. Int J Biol Sci.

[2] Tang PM, Zhang YY, Xiao J, Tang PC, Chung JY, Li J (2020). Neural transcription factor Pou4f1 promotes renal fibrosis via macrophage-myofibroblast transition. Proc Natl Acad Sci U S A.

[3] Tang PM, Nikolic-Paterson DJ, Lan HY (2019). Macrophages: versatile players in renal inflammation and fibrosis. Nat Rev Nephrol.

[4] Orecchioni M, Ghosheh Y, Pramod AB, Ley K (2019). Macrophage Polarization: Different Gene Signatures in M1(LPS+) vs. Classically and M2(LPS-) vs. Alternatively Activated Macrophages. Front Immunol.

[5] Shapouri-Moghaddam A, Mohammadian S, Vazini H, Taghadosi M, Esmaeili SA, Mardani F (2018). Macrophage plasticity, polarization, and function in health and disease. J Cell Physiol.

[6] Sica A, Mantovani A (2012). Macrophage plasticity and polarization: in vivo veritas. J Clin Invest.

[7] Ye S, Huang H, Xiao Y, Han X, Shi F, Luo W (2023). Macrophage Dectin-1 mediates Ang II renal injury through neutrophil migration and TGF-β1 secretion. Cell Mol Life Sci.

[8] Trinath J, Holla S, Mahadik K, Prakhar P, Singh V, Balaji KN (2014). The WNT signaling pathway contributes to dectin-1-dependent inhibition of Toll-like receptor-induced inflammatory signature. Mol Cell Biol.

[9] Suryawanshi A, Tadagavadi RK, Swafford D, Manicassamy S (2016). Modulation of Inflammatory Responses by Wnt/β-Catenin Signaling in Dendritic Cells: A Novel Immunotherapy Target for Autoimmunity and Cancer. Front Immunol.

[10] Montford JR, Furgeson SB (2017). A new CTGF target in renal fibrosis. Kidney Int.

[11] Miao J, Liu J, Niu J, Zhang Y, Shen W, Luo C (2019). Wnt/β-catenin/RAS signaling mediates age-related renal fibrosis and is associated with mitochondrial dysfunction. Aging Cell.

